# Treatment Strategies for Malignancies of the External Auditory Canal

**DOI:** 10.1007/s11864-021-00931-3

**Published:** 2022-02-15

**Authors:** Shixun Zhong, Wenqi Zuo

**Affiliations:** Department of Otolaryngology, the First Affiliated Hospital of Chongqing Medical University, 1 Youyi Road, Yuzhong District, Chongqing, 400016 China

**Keywords:** External auditory canal, Cancer, Squamous cell carcinoma, Surgical management, Radiotherapy

## Abstract

Malignant tumors of the external auditory canal (EAC) are rare tumors in the head and neck. Delayed diagnosis is not uncommon because the symptoms of early tumors are nonspecific. Various surgical and oncological treatment modalities have been reported. Decision-making depends on pathological feature and stage of the lesions, patient’s general condition and preference, and physician’s experience and skill. Radical surgery is widely accepted as the primary treatment of choice. Postoperative radiotherapy is used more often to improve local and regional control of the disease. Chemotherapy is usually recommended for advanced disease, residual disease, and metastasis. Prognosis is affected by multiple factors such as TNM stage, surgical margin, pathological type and differentiation of tumor, involvement of facial nerve, and so on. Although the survival rate is improved significantly over the past several decades with the development of skull base surgery, neuroradiology, anesthesiology, and oncology, it remains challenging to diagnose and treat EAC malignancies due to the rarity, the local anatomical complexity of temporal bone, and the lack of standard TNM staging system.

## Introduction

Malignant tumors of the EAC account for about 0.2% of all head and neck malignancies [1]. The annual incidence is estimated between 1 and 6 per million population [2, 3]. They are more common in male patients aged 60–70 years [4–6]. Anatomically, the temporal bone is not a barrier but a medium for tumor spread through the potential pathways, e.g., the bony canals and intraosseous vessels and nerves, the petrosquamous suture line, the fissures of Santorini, the foramen of Huschke, the stylomastoid foramen, and so on.

The most common histologic type of EAC malignancies is squamous cell carcinoma (SCC), which accounts for 80% of tumors within the temporal bone, followed by other tumors such as basal cell carcinoma, adenoid cystic carcinoma, adenocarcinoma, and so on [7–9]. The following discussion primarily focuses on the SCC.

Because the symptoms of early tumors are nonspecific, diagnosis is usually delayed. It has been reported that 65% of patients are at T3 or T4 at the first visit, and the average time from initial symptom to diagnosis is 12.4 months to 3.9 years [6, 10]. Radiological imaging assessment of head and neck is essential for accurate tumor diagnosis and staging [11]. CT and MRI are complementary imaging examinations for EAC malignancies [12]. Nevertheless, the extent of tumor may be either overestimated or underestimated despite scanning using both CT and MRI [13, 14].

There is no consensus currently on TNM staging of these tumors by the American Joint Committee on Cancer (AJCC) or the Union for International Cancer Control (UICC). The most widely accepted system is the Pittsburgh staging system [1]. Nevertheless, the Pittsburgh staging system is proposed for SCC in the EAC. Though a few authors extrapolated the Pittsburgh classification to other histological types [5, 15, 16], its role in evaluating tumors of other histological types in the EAC or tumors of other subsites of the temporal bone needs to be studied further. The presentation, extension, and treatment of these tumors may be of great difference from those of the SCC in the EAC.

## Treatment

Although various surgical and oncological treatment modalities including surgery, radiotherapy, and chemotherapy have been reported, there is no consensus on management of SCC of EAC.

### Surgery

#### Surgical approaches

Radical surgery is widely accepted as the primary treatment of choice. There are two types of surgical approaches to EAC malignancies, i.e., en bloc and piecemeal resection [17]. An alternative is combination of en bloc and piecemeal resection which is usually used for T4 tumor [9]. Mazzoni et al. have reported that histopathological evaluation of a full specimen achieved by en bloc resection is helpful to establish a correlation between clinical, pathological features and outcomes, so they suggested that en bloc resection should always be the primary consideration in ear carcinoma [18]. Muelleman et al. [19•] showed that there were no differences in overall survival and disease-free survival between en bloc and piecemeal lateral temporal bone resection for the SCC of the EAC. Though there are still controversies on en bloc versus piecemeal resection, the decision should be made based on the status of tumor and experience of surgeon.

#### Surgical techniques

The EAC malignancies can be removed by sleeve resection, lateral temporal bone resection (LTBR), subtotal temporal bone resection (STBR), or total temporal bone resection (TTBR) according to the site and extent of the lesion. The recurrence rate following sleeve resection was reported to be 46.2% (12/26) for T1 and T2 SCC in EAC [20]. Therefore, it is generally believed that LTBR is the primary surgery of choice for T1 and T2 tumors, while sleeve resection is indicated only for low-grade tumor of the cartilaginous EAC skin without bone involvement. Ghavami et al. [21] reported a modified LTBR with sparing of the TM and ossicles in selected SCC patients to preserve the hearing. They demonstrated similar oncologic results to the LTBR and better hearing results in a short term follow-up(8-50 months). STBR is usually indicated as treatment of choice for T3 and T4 tumors, and TTBR is usually advised for advanced T4 tumors. However, TTBR is still a matter of debate because it may cause serious complications without certain increase in survival rates.

#### Parotidectomy

The parotid is susceptible to extension of tumor in the EAC. It has been reported that the incidence of parotid involvement in SCC of the EAC is between 10 and 62% of patients [8]. Though some authors advocate superficial parotidectomy with LTBRs, especially for tumor eroding the anterior wall of the EAC [5, 22, 23], no evidence that routine superficial parotidectomy contributes better survival rate has been reported. At the Gruppo Otologico in Italy, superficial parotidectomy is performed in T2 tumors only when the anterior wall of the EAC is found to be involved, and in all T3 tumors. Total parotidectomy is indicated for T4 tumors and those with involvement of the parotid per se [8]. Mazzoni et al. [24] prefer to superficial parotidectomy in T1 and T2 cases, and total parotidectomy in T3 and T4 cases.

#### Neck dissection

Most authors agree that neck dissection is indicated for advanced tumors or lymph nodes metastasis. The primary controversy is the neck management for N0 and early tumor. The most common lymph nodes involved are those in levels II–III or levels I–II [4, 25]. Various effects of nodal metastasis on survival rate have been reported. Though some authors advocated prophylactic neck dissection for all patients, even for N0 neck [23, 26–28], the evidence for routine neck dissection in N0 neck, especially in T1 and T2 tumors, is not fully identified. At the Gruppo Otologico, neck dissection for N0 neck and low T stage is performed only when the frozen section of the level II nodes is positive for metastasis. In N+ neck, neck dissection including the parotid and lymph nodes in level II is routinely performed [8]. Gidley et al. [22] recommended selective neck dissection (levels II–III) in T1 and T2 tumors. Rinaldo et al. [27] have proposed neck dissection (level Ib, II-V) for all temporal bone SCC even the neck is at N0.

#### Reconstruction

Radical resection of temporal bone tumor may result in various defects. Anterolateral thigh free flap and radial forearm flap are ideal for extensive defects [29, 30]. Musculocutaneous submental flap is an excellent option with reduced operative time, hospitalization duration, and flap revisions, because of its proximity, reliability, ease of harvest, and exceptional color match [31]. Tanaka et al. reported a new method for selecting intraoperative accurate auricle positions in skull base reconstruction for advanced temporal bone carcinoma cases, so that auricle can be preserved as much as possible from a cosmetic and functional perspective [32]. Morita et al. have shown that reconstruction of the EAC using split-thickness skin graft in combination with tympanoplasty can preserve hearing after LTBR for early-stage external auditory canal carcinoma [33]. Other options such as super-thin superficial circumflex iliac perforator flap, laterocervical twisted flap, and so on have also been reported [34, 35].

### Radiotherapy

The role of radiotherapy in low-stage tumors or as a primary treatment has not been fully identified. Though a few papers reported that outcome of radiotherapy alone was not significantly different from that of combined surgery and radiotherapy [36, 37], most authors advise postoperative radiotherapy to improve local and regional control of the disease. It is usually used in T3 and T4 tumors, and in T1 and T2 tumors with bone erosion, perineural invasion, a positive margin, recurrence, nodal metastasis, and extracapsular spread [38]. Only a few authors recommended postoperative radiotherapy for all patients [39]. Cristalli et al. [40] have showed that intraoperative radiotherapy followed by intensity-modulated radiation therapy for advanced external auditory canal and middle ear tumors is effective and safe. A meta-analysis by Oya et al. [41] showed no difference in survival between the surgery-only and postoperative radiotherapy groups in patients with external auditory canal SCC at early stage (stages I ~ II). However, the prognosis following postoperative radiotherapy was better than that following surgery alone in patients at stage I. The authors therefore recommended postoperative radiotherapy for stage I patients at high risk. Koto et al. have reported that the 1-year and 3-year overall survival rate were 70% and 40%, respectively, following carbon ion radiotherapy for advanced SCC of the external auditory canal and middle ear [42]. Murai et al. [43] have demonstrated promising results following stereotactic radiotherapy for patients with ear cancer (≤T3), mainly due to the advantages of improved target coverage with a steep dose gradient and spares surrounding normal tissues.

The usual dose of postoperative radiotherapy is 50 to 60 Gy for T2-T4 tumor [26]. In Pfreundner’s report [44], 54–60 Gy was recommended for tumor with a negative margin, and 66 Gy for tumor with a positive margin. Prabhu et al. [45] proposed 60–66 Gy for tumor with a free margin, and 68–72 Gy for tumor with a positive margin. Hashi et al. advised a dose of 65–75 Gy for inoperable patients as palliative therapy [37].

### Chemotherapy

Chemotherapy is not recommended as a routine therapy but usually used for advanced disease, residual disease, and metastasis as an induction agent in the adjuvant setting [9, 46••]. It has been shown that a combination of cisplatin with 5-fluorouracil may be the best chemotherapeutic treatment with good pain control though it does not raise the survival rate [47]. A meta-analysis by Takenaka et al. demonstrated that preoperative chemo-RT (CRT) might benefit the postoperative survival of patients with external auditory canal SCC and that the survival rate of definitive CRT was comparable with that of surgery [48]. In a report of small series (13 cases), Kitani et al. showed that concurrent CRT with cisplatin and 5-fluorouracil was safe and effective for patients with temporal bone SCC, especially for stage II and III tumors [49]. Some authors have presented that concomitant chemoradiotherapy with a combination of cisplatin, 5-fluorouracil, and docetaxel (TPF) is a safe and promising regimen for locally advanced temporal bone cancer [50, 51]. Benefit from intra-arterial cisplatin infusion in association with RT has also been reported [52, 53]. In a case report, Ebisumoto et al. showed complete response following radiotherapy plus cetuximab, a monoclonal antibody targeting epidermal growth factor receptor, in patients with T4 EAC SCC. It suggested cetuximab plus radiotherapy might be a treatment alternative for patients with advanced temporal bone cancer [54].

## Prognosis

### Predictors

Various prognostic factors have been reported such as TNM stage, surgical margin, pathological type and differentiation of tumor, involvement of facial nerve, and so on [4, 13, 23, 55]. Piras et al. have shown that Pittsburgh Stage or involvement of mastoid, facial nerve, medial wall of the middle ear, temporomandibular joint, and middle fossa dura are the negative prognostic factors [56•]. Morris et al. [5] showed that the 5-year rate of overall survival, disease-specific survival, and recurrence-free survival were 62%, 70%, and 46%, respectively. They found that the surgical margin status and the presence of extratemporal disease were the strongest predictors of survival and recurrence. Masterson et al. [26] revealed that the 5-year disease-specific survival rate was 44% and that nodal status, poorly differentiated squamous cell histology, and carotid involvement were the poor prognostic indicators. Dura mater infiltration was reported to be one of the strongest negative prognostic factors [57]. The diversity of the prognostic factors showed in literatures may be related to the differences between studies in research design and statistical analysis. As a result, no clear conclusion can be drawn at present. Further studies are needed to ascertain more reliable prognostic factors so that the patients at high risk can be treated with more effective and safer primary modalities.

### Recurrence

Most recurrences occur within the first 2 years after primary treatment [5, 22]. Wierzbicka et al. [10] reported that all relapses in their study occurred in T4 cases with a relapse rate of 27.0% and that the mean time from treatment to recurrence was 6.4 months. The median survival time after relapse was 8 months. Morris et al. [5] found that recurrence was common (72%) in T3-4 tumors, and the mean time to recurrence was 13.1 months.

McRackan et al. [6] reported that 18 of 60 patients (30%) recurred at 5.8 months and that recurrence occurred most commonly locally (30.3–44.4%), followed by regional metastasis (0–22.2%), and the distant metastasis (3.0–15.4%). They showed that the recurrence rate of tumors originating in the skin overlying the parotid gland and the EAC (71.4% and 100%, respectively) was higher than those from the auricle/postauricular skin and temporal bone (26.5% and 0.0%, respectively). The recurrence risk was statistically associated with N stage and cervical node involvement. In addition, the recurrence risk was increased in patients with direct spread to the parotid gland and perineural spread. Zanoletti et al. [23] reported a recurrence rate of 44% (18/41) even if pathologically free margins were archived in all patients. Therefore, it is hard to decide the real “free” negative margins.

Patients with recurrence were found more likely to recur again after further treatment [24, 38]. For the recurrent cases, the mean disease-specific survival following nonsurgical palliative treatment was longer than that following palliative surgery (30.6 ± 15.3 months and 16.2 ± 11.3 months, respectively) [58]. Therefore, non-surgical treatment (chemotherapy, radiotherapy, or specialist palliative care) is recommended for patients with advanced recurrent SCC, and salvage surgery only for early recurrences when radicality is still achievable.

## Non-squamous cell carcinomas

### Basal cell carcinoma (BCC)

Though BCC is the second most common tumor of temporal bone, it is much rarer than SCC. A few papers have reported BCC in temporal bone with small cases series, most of which are aggregate date on temporal bone malignancies. It has been shown that BCC may portend a better prognosis but higher risk of recurrence than most other tumors involving the temporal bone and EAC [59, 60].

Breen et al. [61] reviewed recently a large case series of 42 patients with BCC of the temporal bone and external auditory canal. They showed that the most common presenting symptoms were hearing loss (36%) and otorrhea (26%). The overall survival of patients who had undergone surgery elsewhere and presented with facial weakness was worse. Facial nerve was sacrificed in ten patients (24%). The fact that only two patients (5%) developed regional nodal disease indicated lower metastatic potential of BCC compared to SCC in temporal bone. The survival rate of BCC was better than other temporal bone malignancies.

### Adenoid cystic carcinoma (ACC)

Though ACC arising from the EAC are extremely rare accounting for 5–20% of the primary malignancies of the EAC [16], it is the most common type of glandular malignancy arising from the ceruminous gland. The average duration of symptoms is approximately 9.3 months, and the mean tumor size is 1.6 cm [62]. Liu et al. [63] have showed that the peak age is 50–60 years and that females are more prone to being affected than males. ACC has a propensity towards early perineural involvement, which may be the reason why otalgia is one of the most common symptoms. Surgical treatment with en bloc resection followed by postoperative radiotherapy has been recommended by most authors. Surgery alone is usually used for T1 tumors. High rates of occult parotid involvement (59%) indicate superficial parotidectomy during the initial surgery to obtain safe surgical margins [64]. Because the incidence of nodal metastases in ACC arising in the head and neck is relatively low (5.3%), routine nodal neck dissection is not suggested in most patients [65]. The outcome of ACC is favorable with a mean survival of 8.3–10 years, compared to 1.5–4.7 years in other ceruminous carcinoma types. The prognosis is poor in patients with such histopathological features as solid pattern of growth, bone invasion, perineural invasion, positive resection margins, and involvement of surrounding tissues including parotid gland [66].

### Adenocarcinoma

Adenocarcinomas of EAC are excessively rare malignant tumors that originate usually from the ceruminous gland in the EAC. Xia et al. [67] have reported that compared to SCC, adenocarcinoma is more commonly seen in females, and lymph node metastasis is much less, and lung metastasis is much more likely even at early stage. They found by HRCT and MRI that both the cartilaginous and bony parts of the EAC were involved by SCC at T1 and T2 stages, whereas only the cartilaginous part was involved by adenocarcinoma. In addition, both bone and adjacent soft tissue were involved by SCC and adenocarcinoma at advanced (T3 and T4) stages. Their results suggest that the growth patterns of SCC and adenocarcinoma are different at the early stages while similar at the advanced stages.

The treatment modality and prognosis of adenocarcinoma are not yet well understood because most information about these tumors are from case reports. Using a population-based national database in the USA, Ruhl et al. [68] analyzed 22 cases with ceruminous adenocarcinomas in the EAC between 1973 and 2010. The average age at diagnosis is 60–64 years. The most common symptoms are otalgia, mass in the EAC, and hearing loss. Though there is no consensus on the treatment strategies, the authors suggested that complete surgical resection might be adequate in most cases. Postoperative radiotherapy may improve survival in cases whose tumors are not removed completely or with distant metastasis. Nine patients (41%) were still alive with an average survival of 157 months since diagnosis; two patients (9%) died of their malignancies with an average survival of 45 months. It looks like that the prognosis may not be as poor as has been previously thought.

## Conclusion

Treatment options for EAC malignancies are usually determined by the pathological feature and stage of the lesions, patient’s general condition and preference, and physician’s experience and skill. Although the treatment of malignancies of EAC is complex and controversial, the surgery with clear margins, with or without adjuvant radiotherapy, is the primary treatment of choice for the majority of these tumors. Prospective, randomized, controlled, multi-central, or international investigation may enable establishment of widely accepted guidelines. Cooperative work by a multidisciplinary team comprising oto-neurosurgeons, head and neck surgeons, plastic surgeons, oncologists, radiologists, and pathologists is of great importance to benefit patients.

## References

[1] Moody SA, Hirsch BE, Myers EN (2000). Squamous cell carcinoma of the external auditory canal: an evaluation of a staging system. Am J Otol..

[2] Kuhel WI, Hume CR, Selesnick SH (1996). Cancer of the external auditory canal and temporal bone. Otolaryngol Clin North Am..

[3] Arena S, Keen M (1988). Carcinoma of the middle ear and temporal bone. Am J Otol..

[4] Gidley PW, Thompson CR, Roberts DB, DeMonte F, Hanna EY (2012). The oncology of otology. Laryngoscope..

[5] Morris LG, Mehra S, Shah JP, Bilsky MH, Selesnick SH, Kraus DH (2012). Predictors of survival and recurrence after temporal bone resection for cancer. Head Neck..

[6] McRackan TR, Fang TY, Pelosi S, Rivas A, Dietrich MS, Wanna GB (2014). Factors associated with recurrence of squamous cell carcinoma involving the temporal bone. Ann Otol Rhinol Laryngol..

[7] Moffat DA, Wagstaff SA (2003). Squamous cell carcinoma of the temporal bone. Curr Opin Otolaryngol Head Neck Surg..

[8] Prasad SC, D'Orazio F, Medina M, Bacciu A, Sanna M. State of the art in temporal bone malignancies. Curr Opin Otolaryngol Head Neck Surg. 2014; 22(2):154-165 10.1097/MOO.0000000000000035 24573123

[9] Bacciu A, Clemente IA, Piccirillo E, Ferrari S, Sanna M (2013). Guidelines for treating temporal bone carcinoma based on long-term outcomes. Otol Neurotol..

[10] Wierzbicka M, Niemczyk K, Bruzgielewicz A, Durko M, Klatka J, Kopeć T (2017). Multicenter experiences in temporal bone cancer surgery based on 89 cases. PLoS One..

[11] Touska P, Juliano AF (2019). Temporal bone tumors: an imaging update. Neuroimaging Clin N Am.

[12] Wang Z, Zheng M, Xia S (2016). The contribution of CT and MRI in staging, treatment planning and prognosis prediction of malignant tumors of external auditory canal. Clin Imaging..

[13] Xie B, Zhang T, Dai C (2015). Survival outcomes of patients with temporal bone squamous cell carcinoma with different invasion patterns. Head Neck..

[14] Homer JJ, Lesser T, Moffat D, Slevin N, Price R, Blackburn T (2016). Management of lateral skull base cancer: United Kingdom National Multidisciplinary Guidelines. J Laryngol Otol..

[15] Ouaz K, Robier A, Lescanne E, Bobillier C, Morinière S, Bakhos D (2013). Cancer of the external auditory canal. Eur Ann Otorhinolaryngol Head Neck Dis..

[16] Gu FM, Chi FL, Dai CF, Chen B, Li HW (2013). Surgical outcomes of 43 cases with adenoid cystic carcinoma of the external auditory canal. Am J Otolaryngol..

[17] Beyea JA, Moberly AC (2015). Squamous cell carcinoma of the temporal bone. Otolaryngol Clin North Am..

[18] Mazzoni A, Zanoletti E, Marioni G, Martini A (2016). En bloc temporal bone resections in squamous cell carcinoma of the ear. Technique, principles, and limits. Acta Otolaryngol..

[19] Muelleman T, Chowdhury NI, Killeen D, Sykes K, Kutz JW, Isaacson B (2018). Effect of piecemeal vs en bloc approaches to the lateral temporal bone on survival outcomes. Otolaryngol Head Neck Surg.

[20] Zhang T, Li W, Dai C, Chi F, Wang S, Wang Z (2013). Evidence-based surgical management of T1 or T2 temporal bone malignancies. Laryngoscope..

[21] Ghavami Y, Haidar Y, Maducdoc M, Tjoa T, Moshtaghi O, Lin HW (2017). Tympanic membrane and ossicular-sparing modified lateral temporal bone resection. Otolaryngol Head Neck Surg..

[22] Gidley PW, Roberts DB, Sturgis EM (2010). Squamous cell carcinoma of the temporal bone. Laryngoscope..

[23] Zanoletti E, Marioni G, Stritoni P, Lionello M, Giacomelli L, Martini A (2014). Temporal bone squamous cell carcinoma: analyzing prognosis with univariate and multivariate models. Laryngoscope.

[24] Mazzoni A, Danesi G, Zanoletti E (2014). Primary squamous cell carcinoma of the external auditory canal: surgical treatment and long-term outcomes. Acta Otorhinolaryngol Ital..

[25] Sasaki CT (2001). Distant metastases from ear and temporal bone cancer. ORL J Otorhinolaryngol Relat Spec..

[26] Masterson L, Rouhani M, Donnelly NP, Tysome JR, Patel P, Jefferies SJ (2014). Squamous cell carcinoma of the temporal bone: clinical outcomes from radical surgery and postoperative radiotherapy. Otol Neurotol..

[27] Rinaldo A, Ferlito A, Suárez C, Kowalski LP (2005). Nodal disease in temporal bone squamous carcinoma. Acta Otolaryngol..

[28] Moffat DA, Wagstaff SA, Hardy DG (2005). The outcome of radical surgery and postoperative radiotherapy for squamous carcinoma of the temporal bone. Laryngoscope..

[29] Bertelsen C, Simsolo E, Maceri D, Sinha U, Kokot N (2018). Outcomes of reconstruction after temporal bone resection for malignancy. J Craniomaxillofac Surg..

[30] Thompson NJ, Roche JP, Schularick NM, Chang KE, Hansen MR (2017). Reconstruction outcomes following lateral skull base resection. Otol Neurotol..

[31] Howard BE, Nagel TH, Barrs DM, Donald CB, Hayden RE (2016). Reconstruction of lateral skull base defects: a comparison of the submental flap to free and regional flaps. Otolaryngol Head Neck Surg..

[32] Tanaka K, Yano T, Homma T, Tsunoda A, Aoyagi M, Kishimoto S (2018). A new method for selecting auricle positions in skull base reconstruction for temporal bone cancer. Laryngoscope.

[33] Morita S, Nakamaru Y, Homma A, Sakashita T, Masuya M, Fukuda S (2014). Hearing preservation after lateral temporal bone resection for early-stage external auditory canal carcinoma. Audiol Neurootol..

[34] Iida T, Mihara M, Yoshimatsu H, Narushima M, Koshima I (2013). Reconstruction of the external auditory canal using a super-thin superficial circumflex iliac perforator flap after tumour resection. J Plast Reconstr Aesthet Surg..

[35] Aksu AE, Uzun H, Calis M, Safak T (2013). Reconstruction of external auditory canal with a laterocervical twisted (snail) flap. J Craniofac Surg..

[36] Pemberton LS, Swindell R, Sykes AJ (2006). Primary radical radiotherapy for squamous cell carcinoma of the middle ear and external auditory canal-an historical series. Clin Oncol (R Coll Radiol)..

[37] Hashi N, Shirato H, Omatsu T, Kagei K, Nishioka T, Hashimoto S (2000). The role of radiotherapy in treating squamous cell carcinoma of the external auditory canal, especially in early stages of disease. Radiother Oncol..

[38] Dean NR, White HN, Carter DS, Desmond RA, Carroll WR, McGrew BM (2010). Outcomes following temporal bone resection. Laryngoscope..

[39] Leong SC, Youssef A, Lesser TH (2013). Squamous cell carcinoma of the temporal bone: Outcomes of radical surgery and postoperative radiotherapy. Laryngoscope..

[40] Cristalli G, Mercante G, Marucci L, Soriani A, Telera S, Spriano G (2016). Intraoperative radiation therapy as adjuvant treatment in locally advanced stage tumours involving the middle ear: a hypothesis-generating retrospective study. Acta Otorhinolaryngol Ital..

[41] Oya R, Takenaka Y, Takemura K, Ashida N, Shimizu K, Kitamura T (2017). Surgery with or without postoperative radiation therapy for early-stage external auditory canal squamous cell carcinoma: a meta-analysis. Otol Neurotol..

[42] Koto M, Hasegawa A, Takagi R, Sasahara G, Ikawa H, Mizoe JE (2016). Carbon ion radiotherapy for locally advanced squamous cell carcinoma of the external auditory canal and middle ear. Head Neck..

[43] Murai T, Kamata SE, Sato K, Miura K, Inoue M, Yokota N (2016). Hypofractionated stereotactic radiotherapy for auditory canal or middle ear cancer. Cancer Control..

[44] Pfreundner L, Schwager K, Willner J, Baier K, Bratengeier K, Brunner FX (1999). Carcinoma of the external auditory canal and middle ear. Int J Radiat Oncol Biol Phys..

[45] Prabhu R, Hinerman RW, Indelicato DJ, Morris CG, Werning JW, Vaysberg M (2009). Squamous cell carcinoma of the external auditory canal: long-term clinical outcomes using surgery and external-beam radiotherapy. Am J Clin Oncol..

[46] Lovin BD, Gidley PW (2019). Squamous cell carcinoma of the temporal bone: a current review. Laryngoscope Investig Otolaryngol.

[47] Kunst H, Lavieille JP, Marres H (2008). Squamous cell carcinoma of the temporal bone: results and management. Otol Neurotol..

[48] Takenaka Y, Cho H, Nakahara S, Yamamoto Y, Yasui T, Inohara H (2015). Chemoradiation therapy for squamous cell carcinoma of the external auditory canal: a meta-analysis. Head Neck..

[49] Kitani Y, Kubota A, Furukawa M, Sato K, Nakayama Y, Nonaka T (2016). Primary definitive radiotherapy with or without chemotherapy for squamous cell carcinoma of the temporal bone. Eur Arch Otorhinolaryngol..

[50] Shinomiya H, Hasegawa S, Yamashita D, Ejima Y, Kenji Y, Otsuki N (2016). Concomitant chemoradiotherapy for advanced squamous cell carcinoma of the temporal bone. Head Neck.

[51] Shiga K, Katagiri K, Saitoh D, Ogawa T, Higashi K, Ariga H (2018). Long-term outcomes of patients with squamous cell carcinoma of the temporal bone after concomitant chemoradiotherapy. J Neurol Surg B Skull Base..

[52] Sugimoto H, Hatano M, Yoshida S, Sakumoto M, Kato H, Ito M (2015). Efficacy of concurrent superselective intra-arterial chemotherapy and radiotherapy for late-stage squamous cell carcinoma of the temporal bone. Clin Otolaryngol..

[53] Fujiwara M, Yamamoto S, Doi H, Takada Y, Odawara S, Niwa Y (2015). Arterial chemoradiotherapy for carcinomas of the external auditory canal and middle ear. Laryngoscope..

[54] Ebisumoto K, Okami K, Hamada M, Maki D, Sakai A, Saito K (2018). Cetuximab with radiotherapy as an alternative treatment for advanced squamous cell carcinoma of the temporal bone. Auris Nasus Larynx.

[55] Nam GS, Moon IS, Kim JH, Kim SH, Choi JY, Son EJ (2018). Prognostic factors affecting surgical outcomes in squamous cell carcinoma of external auditory canal. Clin Exp Otorhinolaryngol..

[56] Piras G, Grinblat G, Albertini R, Sykopetrites V, Zhong SX, Lauda L (2021). Management of squamous cell carcinoma of the temporal bone: long-term results and factors influencing outcomes. Eur Arch Otorhinolaryngol.

[57] Zanoletti E, Marioni G, Franchella S, Munari S, Pareschi R, Mazzoni A (2016). Temporal bone carcinoma: classical prognostic variables revisited and modern clinico-pathological evidence. Rep Pract Oncol Radiother..

[58] Zanoletti E, Marioni G, Franchella S, Lovato A, Giacomelli L, Martini A (2015). Recurrent squamous cell carcinoma of the temporal bone: critical analysis of cases with a poor prognosis. Am J Otolaryngol..

[59] Gaudet JE, Walvekar RR, Arriaga MA, Dileo MD, Nuss DW, Pou AM (2010). Applicability of the Pittsburgh staging system for advanced cutaneous malignancy of the temporal bone. Skull Base..

[60] Mohs F, Larson P, Iriondo M (1988). Micrographic surgery for the microscopically controlled excision of carcinoma of the external ear. J Am Acad Dermatol..

[61] Breen JT, Roberts DB, Gidley PW (2018). Basal cell carcinoma of the temporal bone and external auditory canal. Laryngoscope..

[62] Crain N, Nelson BL, Barnes EL, Thompson LD (2009). Ceruminous gland carcinomas: a clinicopathologic and immunophenotypic study of 17 cases. Head Neck Pathol..

[63] Liu SC, Kang BH, Nieh S, Chang JL, Wang CH (2012). Adenoid cystic carcinoma of the external auditory canal. J Chin Med Assoc..

[64] Liu H, Zhang Y, Zhang T, Li F, Dai C (2017). Correlation between the pathology and clinical presentations in patients with adenoid cystic carcinoma of the external auditory canal. Head Neck..

[65] International head and neck scientific group (2016). Cervical lymph node metastasis in adenoid cystic carcinoma of the sinonasal tract, nasopharynx, lacrimal glands and external auditory canal: a collective international review. J Laryngol Otol..

[66] Nagarajan P (2018). Ceruminous neoplasms of the ear. Head Neck Pathol..

[67] Xia S, Yan S, Zhang M, Cheng Y, Noel J, Chong V (2015). Radiologcal findings of malignant tumors of external auditory canal: a cross-sectional study between squamous cell carcinoma and adenocarcinoma. Medicine (Baltimore)..

[68] Ruhl DS, Tolisano AM, Swiss TP, Littlefield PD, Golden JB (2016). Ceruminous adenocarcinoma: an analysis of the surveillance epidemiology and end results (SEER) database. Am J Otolaryngol..

